# Automated Time Series Measurement of Microbial Concentrations in Groundwater‐Derived Water Supplies

**DOI:** 10.1111/gwat.12822

**Published:** 2018-09-25

**Authors:** David W. Owens, Randall J. Hunt, Aaron D. Firnstahl, Maureen A. Muldoon, Mark A. Borchardt

**Affiliations:** ^1^ U.S. Geological Survey Upper Midwest Water Science Center Middleton WI 53562; ^2^ U.S. Geological Survey Laboratory for Infectious Disease and the Environment Marshfield WI, 54449; ^3^ Geology Department University of Wisconsin‐Oshkosh Oshkosh WI, 54901; ^4^ USDA‐ARS Laboratory for Infectious Disease and the Environment Marshfield WI, 54449

## Abstract

Fecal contamination by human and animal pathogens, including viruses, bacteria, and protozoa, is a potential human health hazard, especially with regards to drinking water. Pathogen occurrence in groundwater varies considerably in space and time, which can be difficult to characterize as sampling typically requires hundreds of liters of water to be passed through a filter. Here we describe the design and deployment of an automated sampler suited for hydrogeologically and chemically dynamic groundwater systems. Our design focused on a compact form to facilitate transport and quick deployment to municipal and domestic water supplies. We deployed a sampler to characterize water quality from a household well tapping a shallow fractured dolomite aquifer in northeast Wisconsin. The sampler was deployed from January to April 2017, and monitored temperature, nitrate, chloride, specific conductance, and fluorescent dissolved organic matter on a minute time step; water was directed to sequential microbial filters during three recharge periods that ranged from 5 to 20 days. Results from the automated sampler demonstrate the dynamic nature of the household water quality, especially with regard to microbial targets, which were shown to vary 1 to 2 orders of magnitude during a single sampling event. We believe assessments of pathogen occurrence and concentration, and related assessments of drinking well vulnerability, would be improved by the time‐integrated characterization provided by this sampler.

## Abstract 2

*Article impact statement*: Using the automated sampler described here, microbial concentrations in groundwater are shown to vary significantly over short time periods.

## Introduction

Human and animal pathogens are disease‐causing microorganisms that can affect suitability of the water for certain use, and thus deteriorate groundwater resources and related water availability. Fecal contamination by human and animal pathogens, including viruses, bacteria, and protozoa, is a potential human health hazard, especially with regards to drinking water sources (e.g., Borchardt et al. 2012). Various environmental factors (e.g., occurrence of groundwater recharge, pH, temperature, salinity, UV light exposure) influence the occurrence, fate, and transport of pathogens in the subsurface. Moreover, there is a wide range of potential pathogen sources, each with its own timing, magnitude, and pathways of loading. Therefore, pathogen occurrence in groundwater is expected to vary considerably in space and time (e.g., Bradbury et al. 2013). However, even at one location, pathogen characterization in groundwater systems typically requires large volumes of water (e.g., 600 to 1600 L) to be passed through a filter, which makes characterization of temporal variability more difficult. As a result, usually only one filter is collected. A more comprehensive monitoring program that better represents actual system dynamics would include consideration of short‐term and longer‐term system variability.

The objectives of this study were to design, construct, and test an automated sampler suited for hydrogeologically and chemically dynamic groundwater systems. Our design focused on a compact form to facilitate transport and quick deployment to municipal and domestic water supplies. Although this discussion focuses on a subset of possible microbial pathogens and indicators, we believe these methods have applicability to a wide range of microbe types present in groundwater systems.

## Test Site

**Figure 1:**
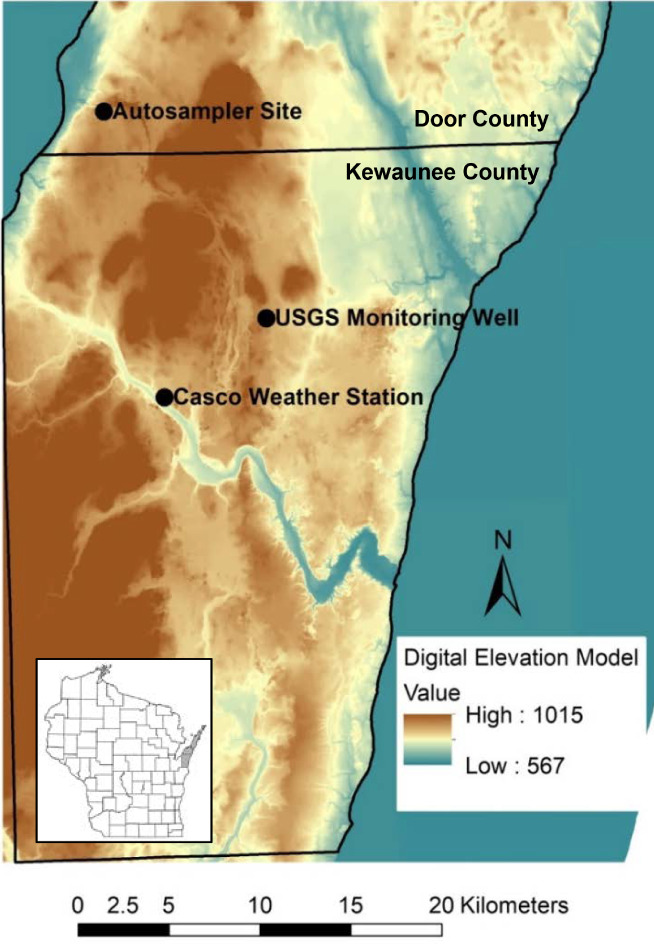
Location of automated sampler location, USGS monitoring well KW183, and Casco, WI weather station plotted on the state digital elevation model (in feet above mean sea level).

The test household was located in Door County, Wisconsin (Figure 1), where the household well obtained water from the upper fractured Silurian dolomite aquifer. This aquifer is a regionally important, but vulnerable, source of drinking water in northeast Wisconsin. In southern Door County, dairy farming and associated crop production comprise the primary land use and manure is commonly applied to crop land. There are no communities large enough to require municipal sewer systems and all homes and commercial enterprises rely on septic systems for on‐site wastewater treatment. This region has a history of bacterial contamination (Sherrill 1978) and occasional “brown‐water” events have also been observed in this area, where household water supplies become temporarily brown in color, odorous, and non‐potable during large recharge events. Because Door County soils are thin, recharge to the dolomite aquifer can be exceedingly rapid and can quickly transport surface contaminants into the aquifer (e.g., Bradbury and Muldoon 1992; Muldoon and Bradbury 2010).

The rapid response of the shallow aquifer is regional. Previous work looking at recharge to the dolomite aquifer (Muldoon and Bradbury 2010) tracked water‐level changes in four wells located in a four‐county area, where all wells had 10 to 20 feet of surficial sediment overlying the dolomite aquifer. Water level changes were correlated between wells, and all wells responded within 24 to 48 h to a large precipitation or snowmelt event. This previous work, and Sherrill (1978)'s observation that bacterial contamination increases during recharge events, supported the monitoring of a non‐pumping “sentinel well” to characterize timing and magnitude of recharge into the larger aquifer system. Groundwater quality after recharge events could then be sampled in domestic household water supply.

This Technical Note describes results from an automated sampler deployed to one household; future publications will focus on the larger recharge characteristics and implications for well vulnerability. The household well sampled during our study was 13.1 m (43 feet) deep and the casing extended to 8.8 m (29 feet) as determined by geophysical and borehole video logs (Peter Chase, WGNHS, personal communication, 2016). The well construction report for a replacement well (installed April 19, 2017) indicates that 6.7 m (22 feet) of surficial sediment overlie the dolomite at this site. The sampler was deployed January 18 through April 9, 2017.

## Microbial Targets

Concurrent with auto‐sampler deployment, a companion study was being conducted in the same region on the sources of fecal contamination in private wells. Four microbial targets were frequently being detected in the companion study: HF183 *Bacteroides*, ruminant *Bacteroides*, pepper mild mottle virus, and rotavirus group A. In the auto‐sampler work, we chose to target these frequently detected microbial markers as well as commonly reported total coliform concentrations.

## Methods

To provide context for automated sampler design decisions, a short description of a commonly employed manual sampling method for pathogens and indicators is provided, followed by a detailed description of the automated sampler workflow. See Appendix S1, Supporting Information regarding the methods used for the microbial analyses.

### Manual Microbial Sampling Protocol

Microbial samples from groundwater are typically collected in two ways. Pumping wells are sampled by connecting a filter to a wellhead tap while the pump was running or piezometers are sampled by peristaltic or other pump with the tubing sterilized between samples. Viruses are concentrated in the field by dead‐end ultrafiltration, which has a pore size of approximately 30‐kDa (Smith and Hill 2009), or glass wool filtration (Millen et al. 2012). Dead‐end ultrafiltration is typically easier to implement in the field and has been used to detect a range of pathogens. Moreover, glass wool filtration requires pH conditioning when groundwater has pH values of 7.5 or higher to ensure virus pathogens adhere to the filter; dead‐end filtration does not have this same requirement. It is recommended that filters be shipped on ice to a laboratory within 48 h of sample collection, where they are backflushed, followed by concentration of the filtrate. The final concentrated sample volume from each filter (typically around 4 mL) is stored at −80 °C until nucleic acid extraction.

### Automated Microbial Sampler

**Figure 2:**
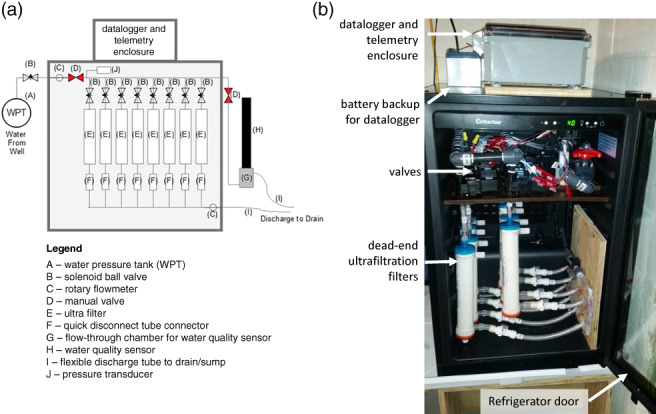
(a) Schematic diagram of automated sampler. (b) Photograph looking into automated sampler refrigerated enclosure.

The automated sampling was performed using a custom‐designed, automated, refrigerated large‐volume virus filtration system (Figure 2). The sampler was installed in the basement of a single‐family dwelling that used a domestic well. The household water supply was tapped at the water pressure tank and fed to an array of dead‐end hemodialysis ultrafiltration filters that were accessed sequentially using solenoid ball valves and datalogger controls. Remote telemetry to the 12‐V battery backed up datalogger allowed operators to monitor current conditions, adjust operational parameters and provide automated data retrieval. Sampling was initiated and monitored using a smartphone. In the work shown here, this design allows sample coverage of entire recharge events (as identified using a United States Geological Survey [USGS] Climate Response Network well; see Figure 1) without locating field personnel at the household site over the course of the whole event.

The sampling system monitored flow rates with several inline rotary flow meters. A flow rate was set using a valve on the system intake; the initial flow rate was set to around 1.2 ± 0.1 L/min. Eight solenoid ball valves were used to sequentially direct flow between the eight dead‐end ultrafiltration filter units. The flow was directed through the filters sequentially based on the volume of water passed through a filter. Target volumes could be changed for the current or future filters during the sampling event. This allows the user to optimize the event sample coverage as the event progresses. A pressure transducer measured backpressure in the filter system, an indication of filter clogging and/or system failure. When a threshold pressure of 345 kPa (50 psi) was met the system would be shut down by closing the main valve into the sampler. If glass‐wool filters were used, the automated sampler design described here could be modified to accommodate pH conditioning (e.g., used by Corsi et al. 2014 for sampling surface water). This sampler used dead‐end ultrafiltration thus pH‐conditioning was not needed. Handling of the filters after collection from the automated sampler followed the same protocol as the manual approach described above.

Water moving through the sampler was also monitored using water‐quality sensors, including temperature, specific conductance, pH, nitrate, chloride, fluorescent dissolved organic matter (FDOM), and turbidity using a multi‐probe water‐quality sonde and a flow‐through chamber. Measurements were made every minute and stored on the sampler's datalogger. Water‐quality data collected were available real‐time using the two‐way telemetry of the sampler. The purpose of this monitoring was to characterize the dynamics and conditions of the household system during time periods when pathogen samples were taken. Because only periodic calibration was performed during the study, reported values are considered to approximate actual analyte concentrations. During initial testing, degassing of the water in the flow‐through chamber caused bubble artifacts in results from the water‐quality sensors (discussed below); orientating the sonde more vertically reduced, but did not eliminate, bubble formation. Water was continually flushed through the flow‐through chamber at a rate of approximately 0.6 L/min. This resulted in a constant relatively low‐flow rate discharge that required constant disposal using gravity drainage into a floor drain. Because the sampler is designed for installation in domestic households it included an emergency shutdown capability triggered by the occurrence of water in the bottom of the sampler. The sampler was constructed using a small refrigerator; therefore, filters could be refrigerated until they were removed to increase holding times.

### Approach for Sampler Testing

Hydrologic conditions used to determine when to activate microbial samplers were tracked using online resources. The regional groundwater system was monitored using real‐time data available from a USGS monitoring well (site number/name: 443535087345401 KW‐25/24E/34‐0183; USGS, 2017).

The local conditions in the household water supply were monitored on a minute time step using low‐flow rates routed through the water‐quality sonde flow‐through chamber. When a target recharge event was identified in the monitoring well hydrograph, the smartphone was used to open the valve to the first filter. The sampler program then monitored the flow rate until the user‐specified volume passed through the filter, at which point the valve to the first filter was shut and the valve to the second filter was opened. This continued until all filters were used or the user canceled the sampling event. Filters were collected every 1 to 3 days during the testing to minimize holding times and maintain continual coverage over the sampling period. During collection, the sampler was paused, filters with quick disconnect couplers were replaced, used filters placed in a plastic bag, sealed, and shipped to the analytical laboratory. Protective latex gloves were worn throughout filter collection to prevent contamination.

## Results and Discussion

Our initial discussion focuses on insights gained from the entire January to April 2017 sampling period; a March to April 2017 snowmelt event within this period is examined in more detail. All water quality data shown here are provided by Owens et al. (2018).

### Entire Sampling Period

#### *Hydrologic Conditions and Sampling Approach*

**Figure 3:**
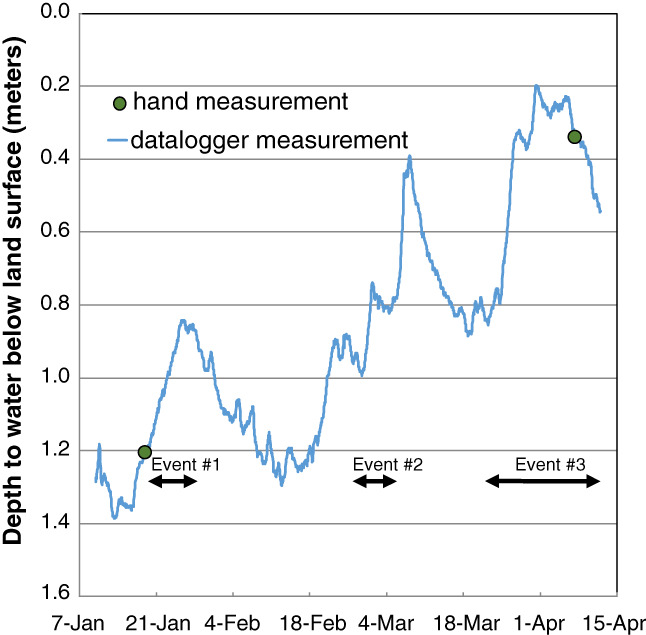
Hydrograph from U.S. Geological Survey Monitoring Well KW183 showing changes in water level during the study period.

Data from the USGS monitoring well shows the period had multiple recharge events (Figure 3). The sampler was run to characterize household water quality during three of these recharge events including: Event #1, had 7 sequential filters collected during a relatively large recharge event January 18 to 23; Event #2, had 8 sequential filters collected during a small recharge event February 28 to March 4; and Event #3, had 26 sequential filters collected during a large recharge event March 21 to April 9. The last of these three events is discussed in detail in the next section. After sampling was initiated, a filter received water until a user‐specified threshold was reached; the amount of water passed through a filter was 750 L (Events #2 and #3) and ranged between 600 and 990 L (Event #1) as the filter collection schedules were being refined. No filter clogging was observed during the study.

#### *Geochemical Time Series*

**Figure 4:**
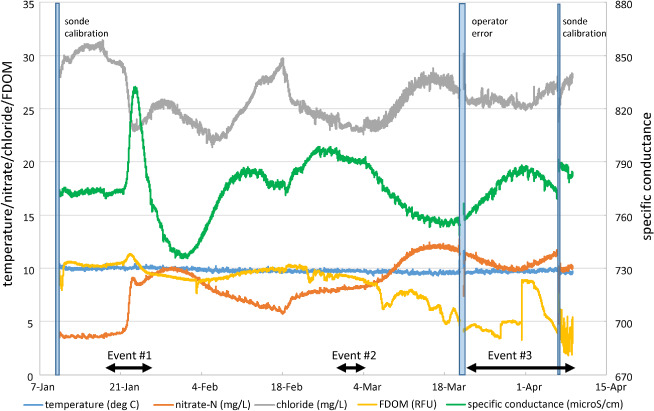
Household water supply quality over the entire study period as measured by the automated sampler sonde (deg °C = degrees Celsius; mg/L = milligrams per liter; RFU = relative fluorescence units; microS/cm = micro‐Siemens per centimeter).

Although the water quality sonde data were only used to provide background characterization and identify potentially anomalous conditions in the household water system, results are included here for illustrative purposes. Water quality measured by the sampler's sonde show fluctuation operating on the order of days and weeks (Figure 4). This is perhaps not surprising because the sonde is sampling a mixed average water quality of the aquifer (i.e., both slow transport/bulk flow and fast transport/low yield contributions that transport pathogens; Hunt et al. 2010). In addition, the effects of recharge in the groundwater system are seen in the geochemical time series when the recharge events are relatively large (e.g., Event #1 and #3; Figure 4). However, individual analytes can move in similar directions, be lagged, or move inversely, likely reflecting the complexity inherent to multiple sources and dual domain transport in the shallow fractured rock aquifer. Water temperature, however, was relatively constant throughout the period the sampler was deployed.

#### *Microbial Water Quality Time Series*

**Figure 5:**
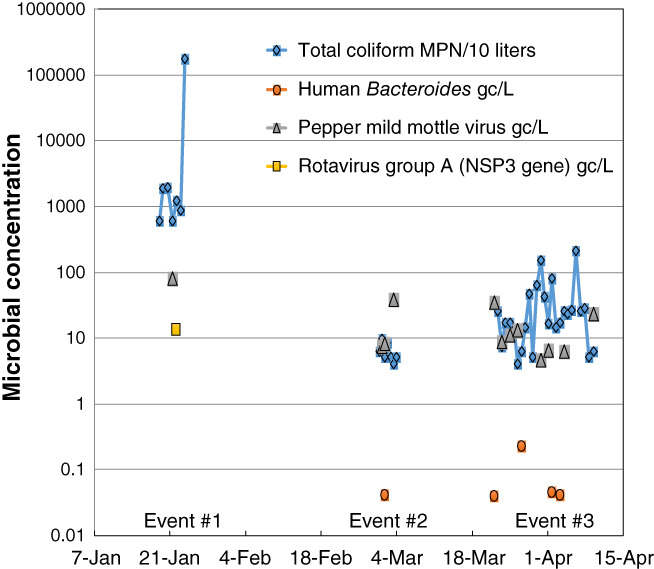
Results of microbial analyses performed on dead‐end ultrafilters collected from the automated sampler during three events. Note all data are plotted on a log scale. All five microbial targets were analyzed for each filter; ruminant Bacteroides was not detected during the sampling. Non‐detections are not shown; when a target organism was absent a gap between time series markers occurs (MPN = most probable number; gc/L = gene copies per liter).

The household microbial water quality was extremely variable, and characterized by appreciable changes in microbial presence/absence and concentration over short periods of time (Figure 5, note the log scale). In all cases but total coliform, microbes analyzed were characterized by presence/absence between filters collected sequentially in time; when present a microbial concentration is shown but when absent a gap between time series markers occurs. Ruminant *Bacteroides* was not found in any sample during the period. Total coliform, found in water from the well in all but one filter, can vary 1 to 2 orders of magnitude during sampling events (Figure 5). It should be noted that the symbols in Figure 5 (and later on Figure 7) reflect the time that a filter was collected for ease of plotting; the value measured for a filter can only reflect the average microbial concentration over the time water passed through the filter.

### March 21 to April 9 Snowmelt Event

#### *Hydrologic Conditions*

A spring 2017 snowmelt period was chosen for detailed description, where the event extended from March 21 to April 9. During this period rain was noted at the Casco, Wisconsin weather station (15 km from the sampler location; Figure 1) during three distinct periods, March 24 to 27, March 29 to 30, and April 2 to 4 (Table 1). All three periods were associated with increasing groundwater levels (Figure 3) in the USGS Climate Response Network monitoring well (14 km away from sampler location; Figure 1).

**Table 1:** Measured Daily Precipitation, Casco, WI (http://www.enviroweather.msu.edu/weather.php?stn=lux)

Date (2017)	Precipitation (inches)	Precipitation (mm)
March 21	0	0
March 22	0	0
March 23	0	0
March 24	0.64	16.3
March 25	0.14	3.6
March 26	0.35	8.9
March 27	0.06	1.5
March 28	0	0
March 29	0.02	0.5
March 30	0.40	10.2
March 31	0	0
April 1	0	0
April 2	0.11	2.8
April 3	0.11	2.8
April 4	0.39	9.9
April 5	0	0
April 6	0	0
April 7	0	0
April 8	0	0

#### *Geochemical Time Series*

**Figure 6:**
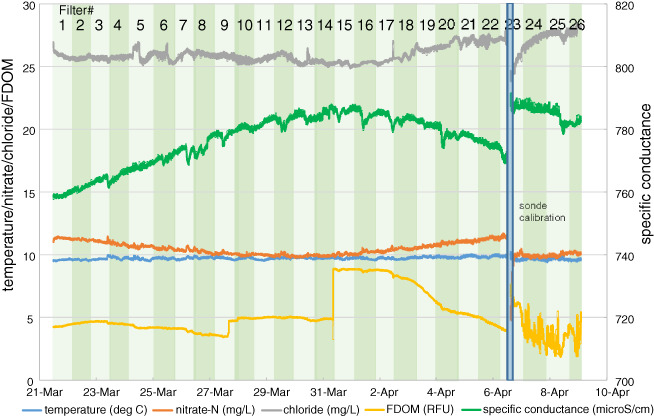
Household water supply quality as measured by the automated sampler sonde during Event #3 sonde (deg C = degrees Celsius; mg/L = milligrams per liter; RFU = relative fluorescence units; microS/cm = micro‐Siemens per centimeter).

Sonde data are summarized as again having broad trends rather than sharp changes (Figure 6). Two trends are apparent during Event #3 that demonstrate an inverse relation between specific conductance and nitrate and chloride; specific conductance increased then decreased over the sampling period while nitrate and chloride decreased then increased during the same period (Figure 6). The lack of synchronicity may reflect enhanced flushing of carbonate‐rich water in the aquifer dolomite matrix and smaller contributions from fractures that contain surface‐derived nitrate and chloride. Temperature was again consistent at or just below 10 °C over the period.

Some water chemistry analyte time series appear to have artifacts from degassing and associated bubble formation/release during this period (e.g., FDOM; Figure 6). Moreover, recalibration of the sensors on the sonde on April 6 also resulted in a change in the sonde reported values (e.g., specific conductance, nitrate; Figure 6), more variable time series after recalibration (FDOM; Figure 6), and an extended recovery period that persisted over a day (chloride Figure 6). As noted previously, these results are perhaps best considered in a more general sense of household water‐quality conditions rather than precise concentrations of the analytes.

#### *Microbial Time Series*

**Figure 7:**
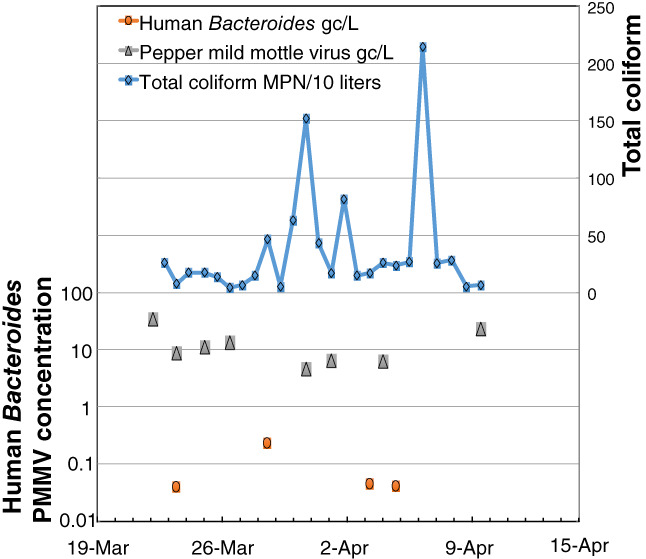
Results of microbial analyses performed on dead‐end ultrafilters collected from the automated sampler during Event #3. Note that total coliform is plotted on an arithmetic scale and human Bacteroides and pepper mild mottle virus (PMMV) are plotted on a log scale. All five microbial targets were analyzed for each filter; ruminant Bacteroides and Rotavirus group A were not detected during the Event #3 sampling. Non‐detections are not shown; when a target organism was absent a gap between time series markers occurs (MPN = most probable number; gc/L = gene copies per liter).

In contrast to the broad trends of the sonde data, time series of total coliform and virus results from 26 sequential filters again show a dynamic system where microbial concentration and occurrence is variable over time, and microbial targets other than total coliform were characterized by presence/absence between filters collected sequentially in time. Moreover, total coliform can vary 1 to 2 orders over the event sampled (Figure 7—note the log scale on the left axis and arithmetic scale on the right axis).

### Best Practices and Potential Limitations

This sampler design was successful in its ability to provide continual sampling coverage of the household water supply. This work was particularly notable for its focus on the more difficult objective of characterizing the water quality of a domestic household water supplies over a multi‐week period; previous research evaluating domestic household wells typically employed a single sampling event to assess pathogens in domestic wells (e.g., Borchardt et al. 2003). From the large variability in time series data, one suggested best practice that results from this work is to limit extrapolation of manual samples of groundwater to a single snapshot of current conditions and not as representing a longer‐term, time‐integrated microbial system.

Best practices for deploying the sampler include four primary topics. First, the field personnel should have uniform procedures that protect against contamination during switching of the filters, such as using color coded filter ends, double bagging, and new sample gloves for each collection trip. Second, it is critical that the flow rates reported by the sampler are accurate because they are used to automate the filter switching and are also used for calculating microbial concentrations. Third, the field personnel should assess the vertical orientation of the water quality sonde to reduce bubble adherence to sensors. And fourth, although refrigeration extends holding times, the sampling schedule should be designed to reduce, to the extent practicable, the time between a filter being filled to when it is processed in the lab.

Current limitations of the sampler design presented here include:

- Long‐term deployment of the water‐quality sonde appeared to result in probe drift that was corrected during recalibration. Therefore, the water quality characterization should be considered qualitative unless more rigorous quality assurance is employed (e.g., more frequent calibration). However, inclusion of a water quality sonde in the sampler provides a cost‐effective geochemical context for the water supply over time. Such insight is important for choosing microbial sampling periods, which helps ensure representative results while lowering the cost of microbial sampling.
- Most household water systems are under pressure, therefore artifacts of bubble formation during depressurization/degassing are expected to occur unless steps are taken to keep the flow‐through chamber pressurized.
- Inadvertent failure to restart the flow to the flow‐through chamber after sonde calibration is not flagged by the datalogger so gaps in the sonde time series can occur (such as seen in March, Figure 4).
- High accuracy in measurement of flows through the system can also be difficult to attain as the flows are near the low end of the operating range of many flow meters. Orienting the flowmeter so the exit point elevation is higher than the entrance point elevation might increase accuracy, and will be tested as part of ongoing sampler improvement.
- Similar to any long‐term water monitoring, the amount of water discharged can be large. At some sites sump pumps or floor drains may not be available. However, the sampler shown here is suitable for deployment outdoors during non‐freezing conditions using an outside spigot.
- When scheduling filter collection to reduce holding times, the number of visits to a site increases. This will increase the cost of field personnel and homeowner disruption.

The sampler also has the more general limitations of microbial characterization in groundwater systems including: lack of information on the source, poor definition of contributing area of the well, and difficulty anticipating transport and lags in transport in the shallow fractured bedrock of the study area. Therefore, longer sampling periods are needed (and more filters/analysis required) to ensure a target system is adequately characterized.

## Conclusions

The results presented here demonstrate that microbe occurrence and concentration time series can be efficiently characterized in the field. These results represent those of shallow fractured aquifers with rapid travel times. In this setting, the sampler documents highly variable domestic household water quality over time. Characterization of groundwater quality using single snapshot sampling can be expected to leave longer‐term characterization of pathogen occurrence and magnitude highly uncertain. Therefore, deploying samplers as described here can be critical for understanding pathogen source and transport, as well as microbial dynamics, in aquifer settings.

## Authors' Note

The authors do not have any conflicts of interest or financial disclosures to report.

## Supporting information


**Appendix S1.** Methods used for microbial sample processing and analysis.
