# Supplementary material for: Automated Time Series Measurement of Microbial Concentrations in Groundwater‐Derived Water Supplies
Source: Ground Water. 2018 Sep 25;57(2):329–36. doi: 10.1111/gwat.12822 (PMC7379695; doi:10.1111/gwat.12822)
Supplement: Supplementary file 1 — Appendix S1. Methods used for microbial sample processing and analysis. [file GWAT-57-329-s001.docx]

**Supporting Information for “Automated time-series measurement of microbial concentrations in groundwater-derived water supplies”**

by

David W. Owens and Randall Hunt; U.S. Geological Survey, Upper Midwest Water Science Center, Middleton, WI, 53562, USA; corresponding author: [rjhunt@usgs.gov](mailto:rjhunt@usgs.gov)

Aaron D. Firnstahl; U.S. Geological Survey, Laboratory for Infectious Disease and the Environment, Marshfield, WI 54449, USA

Maureen A. Muldoon; University of Wisconsin-Oshkosh Geology Department, Oshkosh, WI 54901, USA

Mark A. Borchardt; USDA-ARS, Laboratory for Infectious Disease and the Environment, Marshfield, WI 54449

**Abstract from Associated Technical Note**

Fecal contamination by human and animal pathogens, including viruses, bacteria, and protozoa, is a potential human health hazard, especially with regards to drinking water. Pathogen occurrence in groundwater varies considerably in space and time, which can be difficult to characterize as sampling typically requires hundreds of liters of water to be passed through a filter. Here we describe the design and deployment of an automated sampler suited for hydrogeologically and chemically dynamic groundwater systems. Our design focused on a compact form to facilitate transport and quick deployment to municipal and domestic water supplies. We deployed a sampler to characterize water quality from a household well tapping a shallow fractured dolomite aquifer in northeast Wisconsin. The sampler was deployed from January-April 2017, and monitored temperature, nitrate, chloride, specific conductance, and fluorescent dissolved organic matter on a minute timestep; water was directed to sequential microbial filters during 3 recharge periods that ranged from 5 to 20 days. Results from the automated sampler demonstrate the dynamic nature of the household water quality, especially with regard to microbial targets, which were shown to vary 1-2 orders of magnitude during a single sampling event. We believe assessments of pathogen occurrence and concentration, and related assessments of drinking well vulnerability, would be improved by the time-integrated characterization provided by this sampler.

**Supporting Information**

Sample processing and analysis for microbial targets

Well water was sampled with dead-end ultrafiltration (Smith and Hill 2009) using Hemodialyzer Rexeed-25s filters (Asahi Kasei Medical MT Corp., Oita, Japan); sample volume per filter ranged from 600 to 1000 L. Filters were backflushed using 500 mL of a solution containing 0.01% sodium polyphosphate (NaPP), 0.5% Tween 80, and 0.001% antifoam (Smith and Hill 2009). Desiccated beef extract was added to the backflushed samples at a 1% weight to volume ratio, typically 6.5 g of beef extract into 650 mL of eluate (typically ranging between 500-800 ml). Polyethylene glycol (PEG) 8000 and sodium chloride was used to further concentrate samples following procedures described in Lambertini et al. (2008). Final concentrate sample volumes (FCSV) ranged from 3.2 mL to 5.6 mL. Nucleic acids were extracted from the sample concentrate (from PEG step) using QIAamp DNA blood mini kit with a QIAcube® (Qiagen, Valencia, CA). Reverse transcription (for RNA viruses) was performed using random hexamers (ProMega, Madison, WI) and SuperScript® III reverse transcriptase (Invitrogen Life Technologies, Rockville, MD) following procedures described in Stokdyk et al. (2016). qPCR was used to test samples for human-associated HF183 *Bacteroides* (Green et al. 2014), ruminant *Bacteroides* (Mieszkin et al. 2010), pepper mild mottle virus (Rosario et al. 2009), and rotavirus group A (Zeng et al. 2008). qPCR with hydrolysis probes was performed using a LightCycler® 480 instrument (Roche Diagnostics, Mannheim, Germany) following procedures described in Stokdyk et al. (2016). Standard curves were created from gBlocks® and Ultramer® oligos (Integrated DNA Technologies, Coralville, IA). Lambda phage DNA (New England BioLabs, Ipswich, MA) and hepatitis G virus RNA oligonucleotide (IDT) were used to evaluate all samples for inhibition of qPCR and reverse transcription-qPCR, respectively, following the approach outlined in Gibson et al. (2012). Negative controls were included at all processing steps (secondary concentration, nucleic acid extraction, reverse transcription, and qPCR) and must exhibit no fluorescence above the baseline. Modified live virus vaccines (Zoetis Inc., Kalamazoo, MI) were used for DNA (bovine herpes virus) and RNA (bovine respiratory syncytial virus) extraction positive controls, with the latter serving also as the reverse transcription positive control.

Total coliform analysis was conducted using IDEXX Colilert Quanti-Trays (Westbrook, ME) per manufacturer instructions on a volume of sample concentrate (from PEG step) equivalent to a 10-L sample volume (typically 50 µL of concentrate). Sample concentrates were initially added to 1x phosphate buffered saline (PBS) solution mixed with the IDEXX substrate. The protocol was modified to using reverse osmosis water because we observed that 1x PBS buffer produced lower concentrations than the same sample diluted in RO water. Likewise, sample concentrates were initially stored at -80 °C prior to analysis per our standard laboratory workflow. However, we observed that this single freeze-thaw cycle produced total coliform concentrations lower than expected. The protocol was modified to analyze sample concentrates immediately following the PEG step as both freezing and refrigeration affected measured concentrations. Samples within an autosampler event (i.e., all 8 filters over a single sample period) were treated consistently in terms of analysis matrix (water or PBS) and storage prior to analysis, but the method varied between autosampler events. Therefore, we are cautious in making comparisons between autosampler events, though within-event comparisons are valid.

References

Gibson K.E., K.J. Schwab, S.K. Spencer and M.A. Borchardt. 2012. Measuring and mitigating inhibition during quantitative real time PCR analysis of viral nucleic acid extracts from large-volume environmental samples. *Water Research* 46: 4281-4291.

Green H.C., R.A. Haugland, M. Varma, H.T. Millen, M.A. Borchardt, K.G. Field, W.A. Walters, R/ Knight, M. Sivaganesan, C.A. Kelty and O.C. Shanks. 2014. Improved HF183 quantitative real-time PCR assay for characterization of human fecal pollution in ambient surface water samples. *Appl Environ Microbiol*  80: 3086-3094.

Lambertini E., S.K. Spencer, P.D. Bertz, F.J. Loge, B.A. Kieke BA and M.A. Borchardt. 2008. Concentration of enteroviruses, adenoviruses, and noroviruses from drinking water by use of glass wool filters. *Appl Environ Microbiol* 74: 2990-2996.

Mieszkin S., J.F. Yala, R. Joubrel and M. Gourmelon. 2010. Phylogenetic analysis of *Bacteroidales* 16S rRNA gene sequences from human and animal effluents and assessment of ruminant faecal pollution by real-time PCR. *J Appl Microbiol*. 108, no. 3: 974-984.

Rosario K., E.M. Symonds, C. Sinigalliano, J. Stewart and M. Breitbart. 2009. Pepper mild mottle virus as an indicator of fecal pollution. *Appl Environ Microbiol*. 75, no. 22: 7261-7267.

Smith, C.M. and V.R. Hill. 2009. Dead-end hollow-fiber ultrafiltration for recovery of diverse microbes from water. *Appl. Environ. Microbiol.* 75: 5284−5289.

Stokdyk J., A.D. Firnstahl, S.K. Spencer, T.R. Burch and M.A. Borchardt. 2016. Determining the 95% limit of detection for waterborne pathogen analyses from primary concentration to qPCR. *Water Research* 96: 105-113.

Zeng S.Q., A. Halkosalo, M. Salminen, E.D. Szakal, L. Puustinen and T. Vesikari. 2008. One-step quantitative RT-PCR for the detection of rotavirus in acute gastroenteritis. *J Virol Methods* 153, no. 2: 238-240.

Any use of trade, firm, or product names is for descriptive purposes only and does not imply endorsement by the U.S. Government.

Please note: “Supporting Information” is generally not peer reviewed. Wiley is not responsible for the content or functionality of any supporting information supplied by the authors. Any queries (other than missing materials) should be directed to the corresponding author.
